# Cerebral Wegener's Granuloma: Surgery Mandatory for Diagnosis and Treatment

**DOI:** 10.1155/2013/750391

**Published:** 2013-05-16

**Authors:** Federico Nicolosi, Giovanni Nodari, Giannantonio Spena, Elena Roca, Karol Migliorati, Giacomo Esposito, Roberto Stefini, Marco Fontanella, Pier Paolo Panciani

**Affiliations:** ^1^Division of Neurosurgery, Department of Neuroscience, University of Brescia, Brescia, Italy; ^2^Division of Neurosurgery, Department of Neuroscience, University of Torino, Torino, Italy

## Abstract

The involvement of the central nervous system in case of Wegener granulomatosis (WG) is infrequent and usually leads to cranial nerve abnormalities, cerebrovascular events, and seizures. Meningeal involvement is quite rare and usually is due to the spreading from adjacent disease in the skull base. We described the case of a remote intraparenchymal Wegener's granuloma in a 55-year-old man presenting with seizures and a history of severe generalized WG. The radiological findings were not useful for the diagnosis, and the pharmacological treatment was ineffective. The importance of a surgery in case of localized WG has been emphasized, in order to confirm the diagnosis and to avoid additional medicaments, like antiepileptic drugs, potentially harmful in immunosuppressed patients.

## 1. Introduction

Primitive meningeal involvement represents one of the less frequent manifestations of Wegener granulomatosis (WG) in the central nervous system (CNS). The differential diagnosis is challenging because no radiological findings allow to exclude other extra-axial lesions. Moreover, the pharmacological treatment is often ineffective in case of brain granulomatous lesions [1–5]. We report a case of remote cerebral granuloma in a patient with a history of severe generalized WG. Clinical and radiological features are carefully described and the role of surgery is discussed.

## 2. Case Presentation

A 55-year-old man presented with partial seizures and painful clonus to the right upper limb. He also referred occasional right leg clonus. He was affected by a 17-year history of severe generalized WG with multiple organ involvement (lung, larynx, trachea, kidney, and skin).

### 2.1. Background

WG started 17 years before. It was gradually extended to the lower limbs as purpura and diffuse arthralgia, to the renal parenchyma as extracapillary glomerulonephritis, and to the lung parenchyma with bullous emphysema and diffuse nodularity related to an obstructive ventilator defect. Further granulomas were documented in the glottic region and in the trachea. The patient was subjected to laryngeal nodule excision, lung lobectomy, and tracheal stenting.

Over the years, the patient was treated with cyclophosphamide, azathioprine, and rituximab. On admission he was on a daily maintenance combination therapy with Methotrexate (MTX) (7,5 mg/week) and Prednisolone (5 mg/day).

### 2.2. Neurological Examination

On admission, the neurological examination revealed right mild ptosis and enophthalmos. A mild bilateral pronation and slightly hyper-elicitable reflexes on the right side were also observed.

### 2.3. Laboratory Studies

Blood examination revealed a mild elevation in white blood cells count (11.2 × 103/*μ*L) and iatrogenic hypogammaglobulinemia.

### 2.4. Imaging

**Figure 1:**
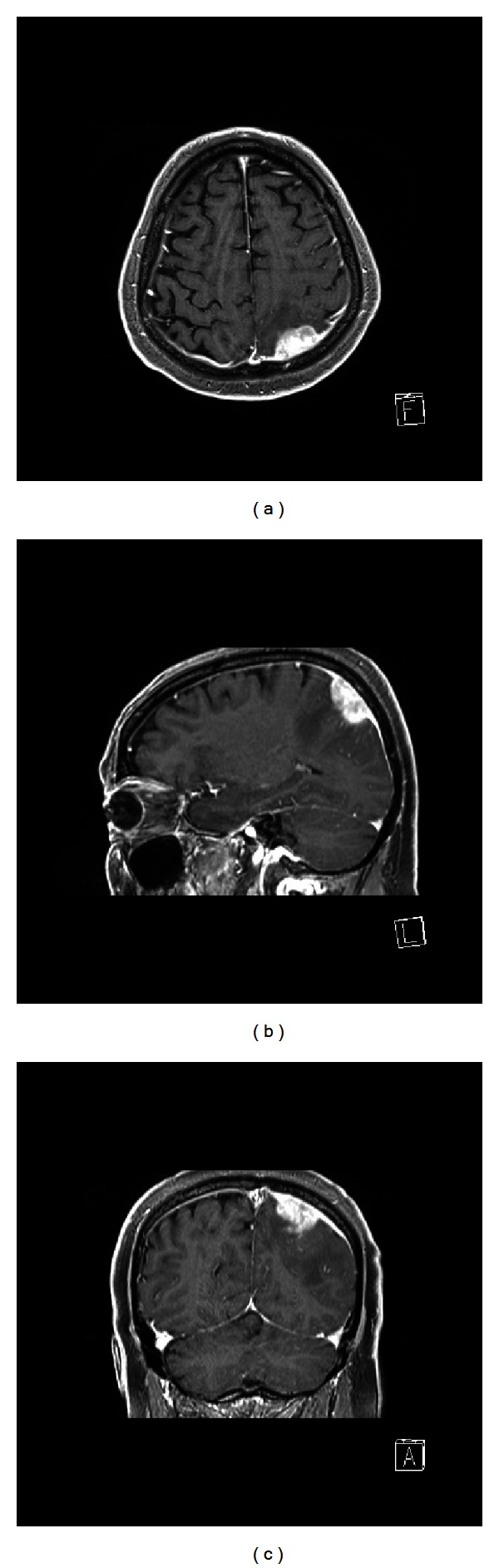
Gd-enhanced, T1-weighted MRI: axial (a), sagittal (b), and coronal (c) view of the brain granuloma; differential diagnosis with other extra-axial lesions is challenging.

The brain CT showed a cortical-subcortical high-density area in the left parietal lobe, with peripheral edema and mass effect on cortical sulci and the ventricular trigone. The brain MRI confirmed the presence of an extra-axial lesion with isohypointensity on T2-weighted sequences and isointensity on T1-weighted sequences. Moreover, the Gd-enhanced MRI showed high enhancement (Figure 1). The lesion had a maximum size of 27 × 16 mm, with irregular boundaries and likely dura mater involvement. A large vasogenic edema due to white matter infiltration was also observed.

### 2.5. Clinical Course

During the hospitalization, a generalized seizure occurred, and the EEG revealed intercritical alterations on the left frontal-temporal region. Antiepileptic therapy was started.

### 2.6. Surgery

**Figure 2:**
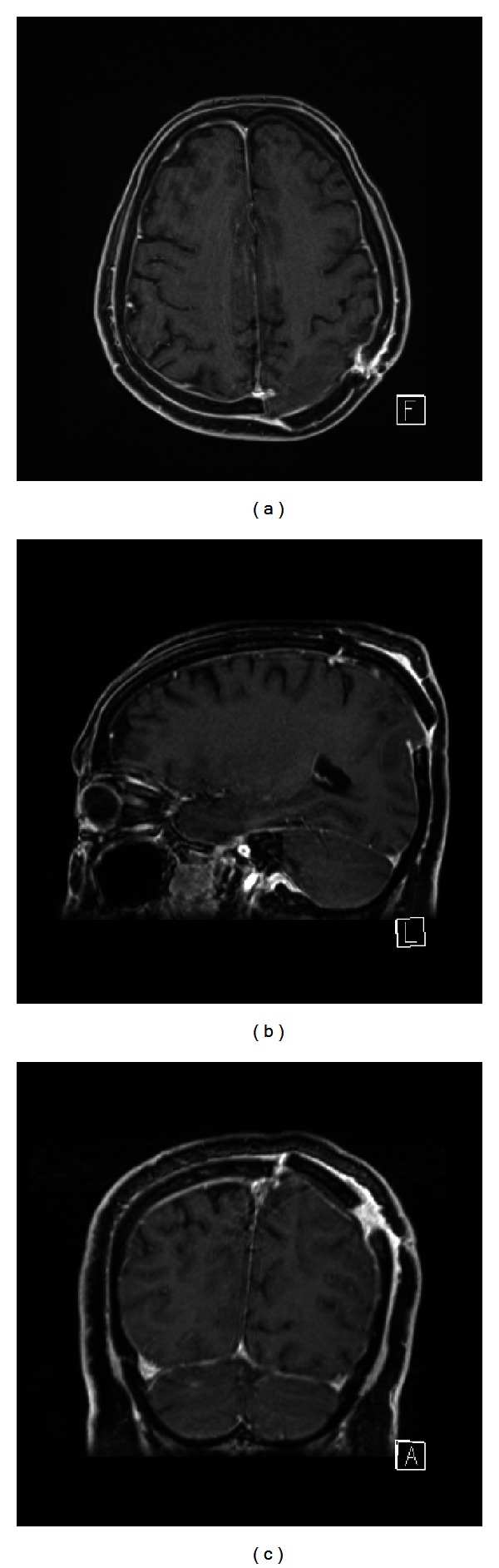
Gd-enhanced, T1-weighted MRI: postoperative axial (a), sagittal (b), and coronal (c) images show the complete excision of the granuloma.

A craniotomy was performed, and the lesion was completely excised. The postoperative MRI confirmed the gross total resection (Figure 2). The postoperative was uneventful, and the patient was discharged after 5 days.

### 2.7. Histological Findings

Macroscopically, the lesion was dark brown, very hard, and poorly demarcated at surgery. The histological examination revealed that it was a Wegner's granuloma. We observed a partly nodular pattern with dural adhesion, containing fibrinoid necrosis phenomena, histiocytes, giant cells, neutrophils, eosinophils, plasma cells, and lymphocytes. Vascular elements included endothelial hypertrophic sleeves with peripheral granulocyte. Negativity of EMA and pancytokeratin markers excluded meningiomas and metastatic lesions.

## 3. Discussion

WG with CNS involvement is the less common form, with a frequency of only 2–6% of all cases [1–9]. Usually WG is related to peripheral nervous system manifestation [10], such as symmetrical polyneuropathy (55% of all cases) or mononeuritic forms (45%) [4]. Principal CNS locations are orbital, nasal, and paranasal areas. From these regions WG infiltrates the adjacent tissues up to meninges or neural structures. Involvement of cranial nerves is also reported [1, 6, 7].

Meninges involvement occurs generally as secondary spreading, in form of thickening and with possible radiological findings of focal or diffuse pattern [8, 11]. Hypertrophic pachymeningitis is more frequent than leptomeningitis (81% and 27%), with higher prevalence in brain than in spinal cord. Local spreading frequently occurs more in the context of localized WG, in which the erosion of the upper airway is often the starting point [11].

Primitive meningeal involvement represents one of the less frequent manifestations in the CNS. In a series of 324 patients, Nishino et al. [1] reported only one case of thickening and enhancement of tentorium cerebelli, while none of the 85 cases described by Fauci et al. [3] meninges involvement was observed. Moreover, remote granulomatous lesions within brain parenchyma have rarely been described [9].

According to the anatomical classification of WG and the Etanercept Trial Research Group functional staging, the WG of our patient was identified as a generalized and severe form [12, 13]. The cerebral localization is a quite rare finding. The lesion resulted in close contact with the dura and infiltrated the underlying parenchyma. No involvement of the orbits, nose, paranasal, or pituitary region was detected. Therefore, we assume that the lesion was an exceptional case of remote intraparenchymal Wegener's granuloma.

Differential radiological diagnosis of the lesion was challenging. According to the radiological features and the immunosuppressive status of the patient, an atypical meningioma and a cerebral abscess were both supposed. In a series of 20 patients, Sherman and Stern identified a possible differential diagnosis of diffuse symmetric meningeal thickenings with primary meningeal tumors [2]. However, the radiological diagnosis of these rare forms of WG is still debated [8].

The patient presented a long history of immunodepression and severe hypoglobulinemia. Therefore, the dosage of maintenance therapy for the WG was reduced in spite to the recommended guideline. As a matter of fact, Prednisolone and Methotrexate were at the lower limit of the indicated range [14]. This may be a likely contributory cause of the brain WG spreading. On the other hand, it should be considered that in a series of localized WG cases, granulomatous lesions resulted frequently resistant to the standard therapy compared with other manifestations. This supports the evidence that CNS is more frequently affected by granuloma than by pure vasculitic lesions [12].

We assumed that the surgery was mandatory both for diagnosis and for mass effect reduction. Moreover, we considered that a long-term antiepileptic therapy should be avoided in a patient with a long history of immunosuppressive drugs. Surgery allowed to completely remove the granuloma, and the antiepileptic drugs were suspended.

## 4. Conclusions

Surgery in case of likely intracerebral Wegener's granuloma located in accessible and no critical areas represents a valid terapeutic strategy. As a matter of fact, the pharmacological treatment may be ineffective, and the radiological findings are not useful to perform an accurate differential diagnosis with atypical dural neoplasms. Finally, surgery may allow to avoid additional drugs, potentially harmful in immunosuppressed patients.
